# Symptoms of Mental Health Conditions and Their Predictors Among U.S. Adults During the First Year of the COVID-19 Pandemic

**DOI:** 10.3390/healthcare13050519

**Published:** 2025-02-27

**Authors:** John Boyle, James Dayton, Randy ZuWallack, Ronaldo Iachan, Deborah Krugipudi, Caitlin Flouton Blanco

**Affiliations:** ICF International, 1902 Reston Metro Plaza, Reston, VA 20190, USA; john.boyle@icf.com (J.B.); james.dayton@icf.com (J.D.); randy.zuwallack@icf.com (R.Z.); ronaldo.iachan@icf.com (R.I.); debbie.krugipudi@icf.com (D.K.)

**Keywords:** anxiety, depressive symptoms, first year of COVID-19 pandemic, PHQ-4, mental health, monthly surveys

## Abstract

**Background/Objectives:** This study examined the impact of the COVID-19 pandemic on mental health among U.S. adults during its first year, using monthly surveys from March to November 2020. **Methods:** The primary outcome was the Patient Health Questionnaire four-item (PHQ-4) measure of anxiety and depressive symptoms. Univarite and bivariate analyses were used to provide foundational understanding of key variables. Parametric and non-parametric correlation analyses were conducted to observe the relationship between COVID-19 impacts or risk factors and the frequency of anxiety/depressive symptoms. A series of regression models were fit to assess the impact of pandemic stressors on PHQ-4 scores. **Results:** There was a statistically significant increase in mean PHQ-4 scores and the proportion of respondents with moderate to severe symptoms (PHQ-4 = 6+) between March–June and July–November 2020. Factors such as fear of contracting the virus, health concerns, and lifestyle disruptions had statistically significant impacts on mental health outcomes; however, these effects were more modest than estimates reported elsewhere. Financial strain, particularly among lower-income households and those experiencing job loss, showed stronger associations with increased anxiety and depressive symptoms, but the overall impact on population-level mental health was limited due to the small proportion severely affected financially. Using regression models, we found that demographic factors and pandemic stressors collectively explained about 21% of the variance in anxiety and depressive symptoms. **Conclusions:** This study provides a nuanced understanding of the pandemic’s mental health impact, suggesting that while certain subgroups were more affected, the overall population level increase in anxiety and depression was less pronounced than previously assumed.

## 1. Introduction

The impact of COVID-19 on mortality and morbidity during the first year of the pandemic years is well documented. By the end of 2020, the United States had over 15 million confirmed or probable cases of COVID-19 and more than 300,000 associated deaths. However, concerns have been raised about the mental health impact of this pandemic [1,2], not captured by disease surveillance systems. Past outbreaks noted psychological distress among affected populations [3]. Aside from threats of illness and death, pandemics produce other stressors known to cause clinically significant psychological distress, including job loss, other economic challenges, food insecurity, barriers to health care and education, and social isolation [4]. In the U.S., more than 26.5 million people filed for unemployment insurance by the end of April 2020. Business and school closings, along with shifts to telework and tele-education, stay at home orders, and other forms of physical distancing, may have also contributed to social isolation. A national survey in June 2020 found 41% of respondents reported struggling with mental health, and 11% reported seriously considering suicide recently [5].

Early studies suggested a significant negative impact of the pandemic on U.S. adult mental health [6]. One study suggested a 3-fold increase in the prevalence of depressive symptoms in the U.S. prior to the pandemic [7]. Another, conducted in April 2020, reported an 8-fold increase in reported serious mental illness compared to the 2018 National Household Interview Survey (NHIS) [8]. A comparison of scores for the 4-item measure of mental health from the Public Health Questionnaire (PHQ-4) in the 2018 NHIS and 2018 Behavioral Risk Factor Surveillance System (BRFSS) with the 2020 Census Household Pulse Survey (HPS) found 3.0-to-5.0-fold elevated relative risk for moderate to serious disease [5]. Using the Census Bureau’s HPS, researchers reported the following: “In the first month of the U.S. COVID-19 epidemic, the prevalence of depressive symptoms was higher than in 2017–2019” [9]. Other reports based on similar comparisons concluded that the pandemic caused substantial increases in anxiety and depression [10,11].

Many early studies relied on comparing pre-pandemic estimates from federal health studies, most commonly the NHIS. However, the absence of equivalent measures before, during, and after the end of the pandemic may make such comparisons problematic [12]. The most important sources of governmental estimates of U.S. population health include the NHIS, the BRFSS, the National Survey on Drug Use and Health (NSDUH), and the National Health and Nutrition Examination Survey (NHANES). Except for BRFSS, these in-person household surveys faced disrupted or altered data collection during the pandemic [13,14]. Since BRFSS was conducted by telephone, data collection could continue during the pandemic, but the PHQ-4 and other mental health measures had been dropped from even optional modules after 2018. The 2020 HPS, introduced in late April 2020 by the Census Bureau to measure household experiences during the COVID-19 pandemic, includes measures of anxiety and depression symptoms. However, the HPS primarily used web data collection, with response rates ranging from 1.3% to 3.8% in its first assessment of depressive symptoms [9]. Some smaller longitudinal studies from non-governmental sources, using the same measures, modes, and samples, have reported higher rates of depression and anxiety in American adults in the early months of COVID-19 compared to the prior period [15].

More recently, Kessler analyzed BRFSS data on adult mental health from March to December 2020 compared to the same months in 2017 through 2019. In the absence of PHQ-4 or similar anxiety/depression measures, they used the number of bad mental health days in the past 30 days, which had good concordance with a PHQ-4 score of 6 or higher (clinically significant anxiety/depressive symptoms). The estimated prevalence of clinically significant symptoms was 0.4 percentage points higher in March to December 2020 compared to 2017 to 2019. “The BRFSS results raise the possibility that 2020 US adult pandemic-associated increases in clinically significant anxiety and depression were much more modest than suggested in previous studies” [6]. Similarly, several meta-analyses of the impact of COVID-19, using pooled data from between 11 and 61 studies, have found statistically significant but small increases in self-reported mental health problems [16]. These small increases in anxiety/depressive symptoms during the pandemic is consistent with the absence of any significant increase in age-adjusted rates of death from suicide in the U.S. between 2018 (14.2 deaths per 100,000 standard population), 2019 (13.9), 2020 (13.5), and 2021 (14.1) [17].

This current study expands on recent studies reporting only a modest increase in mental health symptoms among adults during the pandemic. Specifically, we look at changes in these estimates over the pandemic’s first year, as well as the impact of expected stressors on these estimates during the same period. If measures of clinically significant (moderate to severe) symptoms of anxiety and depression do not substantially increase over the course of the pandemic, then we would expect a relatively limited association between pandemic-related stressors and these symptoms. This hypothesis is tested with data from nine national surveys conducted monthly from March through November 2020 using replicate Census-balanced samples from a large national consumer panel.

## 2. Materials and Methods

### 2.1. Study Design

Data were drawn from a project assessing public attitudes and behaviors during the COVID-19 pandemic. To assess pandemic-associated outcomes monthly, a national non-probability panel was used to conduct surveys in a timely and cost-efficient manner. Respondents, drawn from the MFour mobile panel, comprised approximately two million persons in the U.S. The full panel was not designed as a representative sample but rather as a sampling frame from which geographically and demographically representative samples can be constructed from the panel profile.

For each monthly survey, a national Census-balanced (age, gender, and race) sample of approximately 3000 adult panel members was drawn. An initial invitation was sent to the sample by app notification via cell phone, with reminders (up to 3) to non-respondents over the field period. An incentive of up to USD 4 was offered for survey participation. Approximately 1000 interviews were completed in each wave.

Web-based interviews averaged 18 to 22 min, with 21–33% participation rates. These rates, while much lower than many government health surveys, are comparable, if not higher, than many other population surveys [18]. Although high response rates are desirable to reduce the risk of response bias in survey estimates and increase confidence in the results, research suggests that non-response bias is not necessarily associated with the response rate [19]. This study was reviewed by the ICF Institutional Review Board and received a category 2 exemption (IRB project number 2020-149). This exemption is granted for research (e.g., surveys) that observe or record public behavior.

### 2.2. Survey Measures

The survey invitation and introduction did not mention COVID-19 or the pandemic to avoid non-response bias related to the topic. General health measures were introduced at the beginning of the interview, including our primary measure of mental health, a four-item assessment of current anxiety and depressive symptoms from the Patient Health Questionnaire (PHQ-4) [20]. These items measure symptoms in the last 2 weeks on a four-point scale (0–3)—never, for several days, for more than half of the days, or nearly every day. The PHQ-4 sums the 4-item frequencies for a 0–12 scale, where a score of 6–8 indicates moderate symptoms, and a score of 9–12 indicates severe symptoms [20]. A PHQ-4 threshold of 6+ and a subscale threshold of 3+ are reportedly indicative of probable anxiety and/or depressive disorders [20,21,22]. Therefore, in this study, we consider a PHQ-4 score of 6 or higher to be of clinical significance in the screening of anxiety and depressive symptoms.

Questions related to COVID-19 were introduced later, including a perceived threat to personal, family, and public health; a perceived risk of contracting COVID-19; underlying health conditions that increased the COVID-19 death risk; and the impact on employment, income, and other aspects of their life. The items and wordings for these items were drawn from or adapted from existing surveys, including the BRFSS and published polls. Most dependent variables were structured as four-point ordinal variables, although the risk of getting the virus was also measured as a ratio-level variable ranging from 0 to 100. Finally, respondent demographics included nominal (gender and race) and ordinal variables (age, education, and household income).

### 2.3. Statistical Analyses

We initially conducted univariate and bivariate analyses for key dependent and independent variables. A correlation analysis was conducted using both parametric (Pearson) and non-parametric (Spearman) statistics. Analyses of trends between survey waves were conducted with weighted samples (gender, age, and race/ethnicity) to avoid any differences from demographic non-response bias between waves. We then conducted a series of regression models of potential pandemic stressors on current anxiety and depressive symptoms as measured by PHQ-4 scores.

## 3. Results

A total of 8890 responses were received between March and November 2020. Of the respondents, 74% were white, 51% were male, 32% achieved a high school diploma as the highest level of education, 27% were within the 35–49 age group, and 26% had a household income below USD 25,000.

### 3.1. Current Mental Health Symptoms

Our primary measure of current mental health outcomes is based on the frequency of anxiety and depressive symptoms in the past two weeks. These four items are scored as 0–3, and the PHQ-4 is an additive scale of symptom frequency ranging from 0 to 12 (Cronbach’s alpha α = 0.91). The PHQ-4 includes two subscales for anxiety disorder (GAD-2; α = 0.86) and depression (PHQ-2; α = 0.89).

The overall mean score for the PHQ-4 in this sample was 3.37 for the nine monthly waves in 2020, ranging from a low of 3.20 in late March to a high of 3.58 in July, representing a slight linear trend upwards over the course of the nine months (*p* = 0.054; Table 1). The PHQ-4 scores are generally lower in the first four months of the pandemic compared to July–November 2020. If we compare the March–June scores (3.28) to the July–November scores (3.44), there is a small (4.9%) but statistically significant (*p* = 0.029) increase in estimates of current anxiety and depressive symptoms over the course of the year.

**Figure 1:**
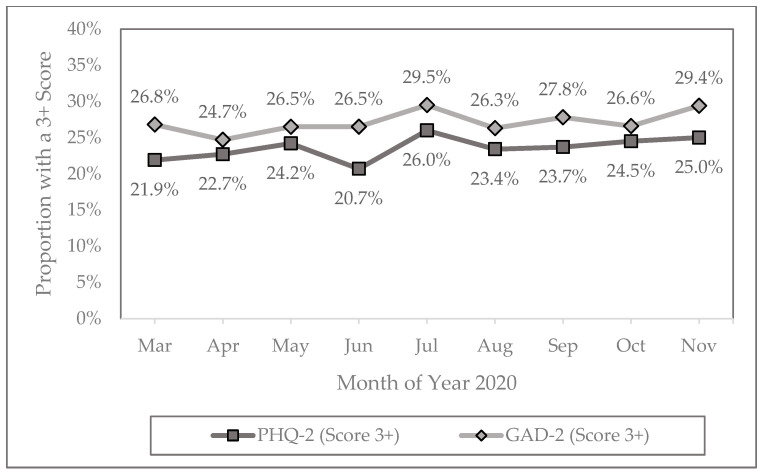
Proportion of PHQ-2 and GAD-2 scores of 3 or higher from March through November 2020. PHQ-2, Patient Health Questionnaire two-item assessment of depressive symptoms; GAD-2, Generalized Anxiety Disorder two-item assessment of anxiety.

Nearly half of the respondents (49.4%) would be classified as having no symptoms of anxiety or depression in the previous two weeks (PHQ-4 score of < 3) during the pandemic. The proportion reporting no symptoms declined from 51.3% in March to 48.6% in November, representing a slight downward trend (*p* = 0.088). Nearly a quarter (24.2%) would be classified with clinically significant symptoms in the previous two weeks (PHQ-4 score of > 5). The proportion reporting moderate to severe symptoms increased from 23.0% in late March to 25.7% in late November, a slight upward trend (*p* = 0.059). If we compare the PHQ-4 results in the early months of the pandemic (March–June) to the later months (July–November), we find a small decrease in the proportion with no symptoms (50.8% to 48.4%) and a small increase in those with clinically significant symptoms (22.9% to 25.3%) (Table 2), which is statistically significant (*Χ*^2^ = 9.24; *p* = 0.026). Like the PHQ-4, both subscale measures, focusing on a score of 3 or higher, increased slightly from March to November 2020, with PHQ-2 rising from 21.9% to 25.0% (*p* = 0.076) and GAD-2 rising from 26.8% to 29.4% (*p* = 0.109; Figure 1).

### 3.2. Clinically Significant Anxiety and Depressive Symptoms by Demographics

This study finds demographic differences in current anxiety/depressive symptoms in the sample during the pandemic’s first year. Females (27%) were more likely than males (22%) to have current anxiety/depressive symptoms (Table 3). The prevalence of current symptoms decreased across age groups, from 33% for 18–24 to 12% for 65 and older. Current symptom prevalence decreased with higher educational attainment, from 39% for less than high school to 17% for college graduates. Symptom prevalence declined from 34% for incomes under USD 25,000 to 15% for over USD 100,000. Hispanics (27%) were more likely than non-Hispanics (24%) to have current symptoms, but no significant white vs. non-white or black vs. non-black differences existed.

### 3.3. Association Between Pandemic Stressors and Current Mental Health Symptoms

This survey included likely pandemic stressor measures, including illness with COVID-like symptoms, a perceived threat of the virus to the respondent and their family, and loss of jobs or household income from the pandemic. There was only a modest association between PHQ-4 scores and having been sick with what might be COVID-19 (0.217). The association between PHQ-4 scores and the perceived likelihood of getting sick with COVID-19 on a scale of 0 to 100% (0.188) or a four-point semantic scale (0.169) was also modest. The correlation between current symptoms and an underlying health condition that would increase the risk of dying from COVID-19 (0.143) was also slight. All these differences are statistically significant at the 0.01 level (Table 4).

The relationship between current anxiety/depressive symptoms and worries about family members catching COVID-19 (0.199) is also modest. The relationship is even weaker with concerns about the spread of COVID-19 in their community (0.132). Concerns about the availability of resources of local hospitals to treat all patients with COVID-19 (0.180) is also modest. All these differences are statistically significant at the 0.01 level.

Among the labor force, the survey also finds a very modest correlation between current anxiety and depressive symptoms and measures of employment and income loss during the pandemic. The correlation between current PHQ-4 scores and employment changes since the beginning of the COVID-19 pandemic is 0.150 for permanently laid off, 0.053 for temporarily laid off, 0.112 for reduced work hours, 0.078 for salary of hourly rate reduced, and −0.164 for none of these. Similarly, the correlation between PHQ-4 scores and changes in current household income compared to the end of 2019 was −0.156. The strongest relationship with current anxiety and depressive symptoms was found with two food insecurity items added later in the pandemic for all respondents: worried whether food would run out before we got money to buy more (−0.331) and the food we bought didn’t last, and we didn’t have money to get more (−0.322).

### 3.4. Pandemic Predictors of Current Mental Health Symptoms

**Figure 2:**
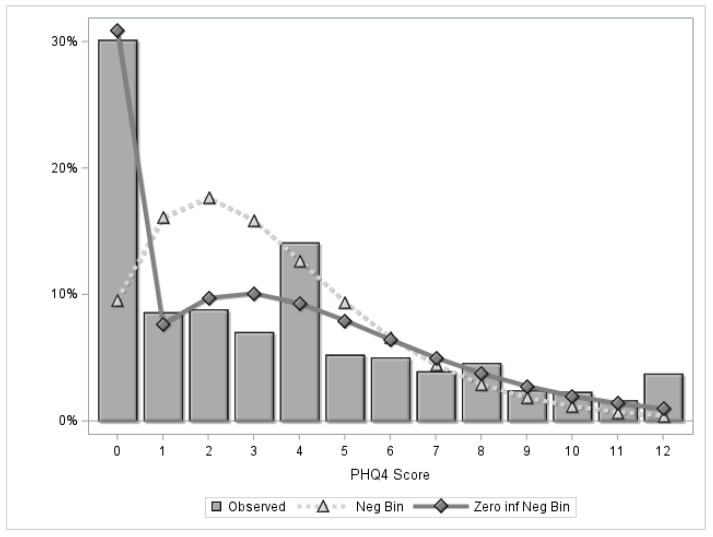
Frequency distribution of PHQ-4 scores with model-based estimates of the negative binomial and zero-inflated negative binomial distributions. PHQ-4, Patient Health Questionnaire four-item assessment of anxiety and depressive symptoms; Neg Bin, negative binomial; Zero inf, zero inflated.

A series of regression models examining the relationship of potential pandemic stressors on current anxiety and depressive symptoms (PHQ-4) were then conducted. Linear regression initially modeled the correlation between the stressors and average PHQ-4 score. However, the distribution of the PHQ-4 scores include nonnegative integers ranging from 0 to 12, with a high number of zero scores. Therefore, we also modeled the data using a negative binomial model to improve estimates of mean PHQ-4 scores for the stressor categories. Figure 2 includes the observed distribution of PHQ-4 scores and model-based estimates using a negative binomial and a zero-inflated negative binomial distribution. To accommodate the excess number of zeros in the distribution, we used a zero-inflated model, which combines a negative binomial regression model of PHQ-4 scores with a logistic regression model estimating the probability of reporting a zero score.

In the first model, we used only demographics reported in the literature that were associated with anxiety and depression. These demographic predictors were gender, age, Hispanic ethnicity, race, education, and household income. The linear, negative binomial and zero-inflated negative binomial regression results for Model 1 are presented in Table 5. Positive values of the negative binomial parameter estimates indicate higher levels of PHQ-4 scores relative to the reference group. Negative values of the zero-inflation parameter estimates indicate a higher likelihood of reporting a positive PHQ-4 score. The model components were combined to estimate the mean PHQ-4 score conditional on the predictors. To aid in the interpretation of the model, we calculated the adjusted mean PHQ scores for each characteristic. All demographics were significant predictors of PHQ-4 scores, with higher levels of current anxiety/depressive symptoms for young adults (4.17 and 4.07 for ages 18–24 and 25–34, respectively); individuals with a less than USD 25,000 household income (4.05); and individuals with less than a high school education (4.03).

In Model 2, we added stressors that had been included in all nine waves from March through November 2020 to the demographic predictors. These stressors included the following: personally diagnosed with COVID-19, other household member diagnosed, concerned about virus spread in your community, worried about family members getting COVID, how likely you were to get COVID-19 (semantic scale), how likely you were to catch the virus (0–100%), presence of an underlying condition making one more likely to die from the virus, how much your life had been disrupted by the virus, and worry whether your local hospital had the resources to treat all patients (Table 6). For brevity, the demographic parameters are suppressed, while the full models are presented in the Appendix A (Table A1). All the stressors were significant predictors of PHQ-4 scores. The highest contrast occurred between people who reported that their life was disrupted by the coronavirus a lot (3.77) compared to those who reported not at all (2.24). The highest adjusted mean scores occur for those reporting that they or a family member had COVID-19 (4.75–5.29) and those who have an underlying heath condition (3.91). The addition of these stressors improved the model fit.

The third model included variables measuring whether the respondent had been laid off permanently or temporarily or had work hours reduced as a result of COVID. These questions were only asked of respondents in the labor force at the beginning of 2020. As expected, people who had experienced permanent loss of employment had higher PHQ-4 scores (3.95), followed by reduced pay or work hours (3.50) and temporary unemployment (3.48) relative to those who experienced none of these (2.93; Table 7).

A fourth regression was conducted with two additional stressors which had been added in the second wave of the survey (asked from April through November 2020): difficulty in paying rent (no mortgage/rent was recoded to ‘no trouble’) and whether they had been sick 3 days or longer when they thought they had COVID-19. The loss of the first wave reduced the sample size to 7512 cases. Those who reported a lot of difficulty had an adjusted mean of 4.64, much higher than those reporting no difficulty (2.69; Table 8). Those who were sick for 3 days and thought they had COVID-19 had an adjusted mean of 3.95, higher than those who did not (3.11). Complete results for Models 3 and 4 can be found in the Appendix A (Table A2).

## 4. Discussion

This study of the impact of the pandemic on mental health among U.S. adults is unusual, because it is based on monthly measures from March through November 2020 of current anxiety and depressive symptoms with an extensive set of pandemic-related stressors. The findings suggest that the mean score for current anxiety and depression and the proportion of respondents with moderate to severe symptoms (clinically significant level) increases only slightly but significantly between March–June and July–November during first year of the pandemic.

The range of subscale, PHQ-2 and GAD-2, estimates over the nine months are similar in magnitude to anxiety and depression estimates produced from the CDC Pulse Survey and the COVID-19 States Project during this time [12] (p. 14). Across subscales and the PHQ-4 scale, Hispanics were more likely than non-Hispanics to have current symptoms, but no significant white vs. non-white or black vs. non-black differences existed. Our findings are consistent with pre-pandemic mental health literature patterns across demographics [23,24,25,26,27]. These findings are consistent with Kessler et al., who found that the estimated prevalence of clinically significant anxiety and depression using the HRQL mental health days from BRFSS was only modestly higher in March to December 2020 compared to March to December from 2017 to 2019 [12]. While our measure of mental health, samples, and mode of data collection differ from Kessler et al., our data agree that while mental health symptoms, including anxiety and depression, increased during the first year of the pandemic, these increases were much more modest than suggested in some earlier studies [12,28,29].

More importantly, this study provides a potential explanation for why this pandemic may have had less of an impact on adult mental health than expected. Our regression analyses suggest that pandemic-related stressors had only a modest impact on anxiety and depressive symptoms during the first year of the pandemic. Our final regression found that eight potential pandemic-related stressors did have a significant impact on current anxiety and depressive symptoms in these national adult samples. The findings suggest that fears of contracting the disease and its impact on health and lifestyle produced a statistically significant impact on adult mental health outcomes, but that impact was more modest than initially suggested in early studies [30,31].

There is evidence of an increase in anxiety and depressive symptoms related to financial strain, particularly for those with lower household incomes. People in the workforce tended to have the highest PHQ-4 scores, with older adults and retirees having very low scores. Literature reviews of earlier studies found that those unemployed as a result of recession were more likely to suffer from stress, anxiety, and depression [32,33,34]. Similarly, our study found that those who were impacted financially by the pandemic had higher levels of anxiety and depressive symptoms, including those who lost a job or struggled to pay for housing. While not included in the modeling due to limited data, the strongest correlations between stressors and current mental health outcomes were job loss and food insecurity. We anticipate that if sufficient data had been available to include food insecurity in the model, they would have shown a significant positive association with PHQ-4 scores, as demonstrated in a study by Coley et al. [35].

The impact of financial strain is muted in the trend analysis from March to November, largely due to the low percentages of people who experienced job loss or had considerable difficulty paying for housing. The percentage of people who had been terminated during the pandemic increased from 4% in March to about 8% in November; though doubled, this is still a small percentage of the population. According to the Current Population Survey, national estimates of unemployment were 4.4% in March of 2020 and 6.7% in November, which is similar to the rates we observed [36]. Further, our study found that the proportion of Americans who struggled a lot to pay for housing stayed fairly consistent between March and November, generally ranging from 10 to 15%. However, the HPS national estimates of housing insecurity, meaning the respondent missed a payment or had low confidence in their ability to pay the next bill, had greater variability during this period, ranging from 7% to 27% [37]. Participants in our study who suffered financially were more likely to experience anxiety and depressive symptoms, but the proportion was small enough that it had little impact on the overall population average.

Although binomial regressions best model these data, our initial linear regressions provide a measure of the variance explained by each model (*R* square) for a simpler comparison of the impact of demographics and stressors in predicting current anxiety and depressive symptoms (PHQ-4). The first model with demographic predictors had an *R* square of 0.085. In the second model, with both demographic and pandemic stressors from all nine waves, we obtained an *R* square of 0.159. The third model included the three additional employment variables: laid off permanently, temporarily laid off, and work hours reduced. Those not employed at the beginning of 2020 were treated as if these events had not occurred to them. Only being terminated was significant but had a trivial impact on the model’s *R* square of 0.161. The fourth regression was conducted with the additional stressors of difficulty in paying rent/mortgage and being sick with what they thought was COVID-19, which reduced the sample size (no first wave) but increased the *R* square to 0.210. A large multi-country study conducted by Fountoulakis et al. included many of these variables and observed similarly low *R* square values when used to predict a change in anxiety (*R* square = 0.164), change in depressive symptoms (0.135), and the development of distress or depression (0.239) [38]. Therefore, we conclude that many potential pandemic stressors are statistically significant predictors of anxiety/depressive symptoms, but collectively, these stressors contribute very little to the total variance in PHQ-4 scores during the pandemic.

### Limitations

To conduct monthly national assessments of pandemic stressors and health outcomes, this study used a demographically and geographically representative non-probability consumer panel. This approach permitted us to gather information about current mental health symptoms and pandemic-related stressors monthly among demographically representative samples in a cost-effective manner. We are unaware of any other probability-based survey collecting both mental health measures and an equally comprehensive set of pandemic-related stressors across this same period.

Projections of sample estimates to populations within statistical limits cannot be made from non-probability samples, and participation rates are substantially lower than gold standard federal surveys [39,40], though comparable for most other surveys [41]. While survey estimates from non-probability surveys are not necessarily biased or unrepresentative, it is reasonable to be concerned about how a low response rate and non-probability selection might affect survey representativeness [42]. As a result, inferences drawn from this study may be limited due to the sample design and the notation that adults who chose to participate may be different from those who declined to respond. Conversely, since we did not have to disclose the survey topic or sponsorship or make appeals to social utility, the panel approach avoided some common sources of potential bias from probability samples.

We also note that the measures of change were limited by the repeated cross-sectional nature of the design. Unlike a longitudinal design, changes in employment and income could only be assessed by respondent reports at one point in time. It is significant, however, that these measures showed a strong association with mental health outcomes (Section 3.3 and Table 7 and Table 8). Moreover, assessments through *R* square need to be qualified with the fact that some of the unexplained variances may be due to psychosocial factors not measured in this study.

Finally, our primary goal was to examine the impact of pandemic-related stressors on mental health symptoms during the pandemic, not point estimates of these stressors or current mental health in the population. Although probability samples are the gold standard for survey research, non-probability samples are increasingly accepted when factors such as a limited budget, high data collection costs, or urgency make it infeasible to use a probability sample [43,44]. Hence, we believe that this study meets the “fit-for-purpose” criteria in monitoring public health trends during a crisis, particularly when there are no probability samples providing equivalent data. Moreover, due to the relatively inexpensive and rapid data collection that this method offers, it appears to be a good way to identify key findings and trends that could be followed up with more traditional probability sampling and data collection procedures, as recommended in the survey methodology literature [45,46].

## 5. Conclusions

The current study explored the rate of mental health symptoms in the US adult population during the first nine months of the COVID-19 pandemic (March through November 2020) and the impact of a broad range of pandemic related stressors on those outcomes. Specifically, we looked at changes in these estimates of anxiety and depressive symptoms using the PHQ-4 scale over the pandemic’s first year, as well as the impact of demographics and expected stressors on these estimates during the same period. Consistent with more recent studies, we found the mean score for current anxiety and depression in our samples, and the proportion of respondents with moderate to severe symptoms, increased only slightly but significantly between March–June and July–November of 2020. More importantly, this study provides an explanation for why the COVID-19 pandemic may have had less of an impact on adult mental health than expected. Our regression analyses suggest that pandemic-related stressors had only a modest impact on anxiety and depressive symptoms. For comparison purposes, our first regression model with only demographic predictors had an *R* square of 0.085, while our second model with both demographic and pandemic stressors increased the *R* square to 0.159. A third model including additional employment stressors, permanently or temporarily laid off, and work hours reduced, only increased the *R* square to 0.161. While not included in the modeling due to limited data, the strongest correlations between stressors and current mental health outcomes were job loss and food insecurity. The impact of financial strain is muted in the trend analysis from March to November, largely due to the low percentages of people who experienced job loss or had considerable difficulty paying for housing. Therefore, we conclude that while many potential pandemic stressors are statistically significant predictors of anxiety/depressive symptoms, collectively these stressors contributed very little to the total variance in PHQ-4 scores during the early stages of the pandemic. The relatively small increase in mental health symptoms among US adults in the first nine months of the pandemic is attributable to the relatively small subsample of the population affected by the most stressful outcomes, long term job loss and food insecurity. The other pandemic stressors had a relatively modest impact on adult mental health outcomes in our repeated Census balanced national samples from March through November 2020. 

## Figures and Tables

**Table 1:** Descriptive summary of PHQ-4 by time period in 2020.

	PHQ-4 Mean (SD)
March–June (n = 3988)	3.28 (3.39)
July–November (n = 4990)	3.44 (3.52)
Total (n = 8979)	3.37 (3.47)

PHQ-4, Patient Health Questionnaire four-item assessment of anxiety and depressive symptoms; SD, standard deviation.

**Table 2:** Crosstab results of PHQ-4 categories by time period in 2020, including count and weighted percentages.

PHQ-4 Category (Score Range)	March–June Count (Wt %)	July–November Count (Wt %)	Total Count (Wt %)
None (0–2)	2025 (50.8%)	2413 (48.4%)	4438 (49.4%)
Mild (3–5)	1050 (26.3%)	1315 (26.4%)	2365 (26.3%)
Moderate (6–8)	508 (12.7%)	734 (14.7%)	1242 (13.8%)
Severe (9–12)	405 (10.2%)	528 (10.6%)	933 (10.4%)
Total	3988 (100.0%)	4990 (100.0%)	8978 (100.0%)

PHQ-4, Patient Health Questionnaire four-item assessment of anxiety and depressive symptoms; Wt, weighted.

**Table 3:** Weighted proportion of anxiety and depressive symptoms by demographics between March and November 2020.

			GAD-2		PHQ-2		PHQ-4	
			Clinically Significant (Wt %)					
Demographic		N	No	Yes	No	Yes	No	Yes
Total		8980	76.5	23.5	72.9	27.1	75.8	24.2
Gender	Male	4353	77.8	22.2	75.8	24.2	78.5	21.5
	Female	4607	75.4	24.6	70.2	29.8	73.4	26.6
	*p*-value		0.006		0.001		0.001	
Age	18–24	1128	68.1	31.9	61.6	38.4	67.1	32.9
	25–34	1593	70.0	30.0	64.2	35.8	69.0	31.0
	35–49	2410	73.7	26.3	69.8	30.2	72.3	27.7
	50–64	2122	79.1	20.9	77.9	22.1	79.3	20.7
	65+	1726	88.5	11.5	86.3	13.7	88.3	11.7
	*p*-value		0.001		0.001		0.001	
Education	Less than HS	712	59.6	40.4	59.2	40.8	60.7	39.3
	HS Grad	2842	74.5	25.5	70.8	29.2	73.0	27.0
	Some College	2669	76.2	23.8	72.0	28.0	75.5	24.5
	College Grad	2684	83.4	16.6	79.4	20.6	82.7	17.3
	*p*-value		0.001		0.001		0.001	
Household Income	<USD 25,000	2354	67.1	32.9	63.2	36.8	66.1	33.9
	USD 25 k–USD 34,999	1416	72.8	27.2	69.8	30.2	72.9	27.1
	USD 35 k–USD 49,999	1336	80.3	19.7	76.9	23.1	79.6	20.4
	USD 50 k–USD 74,999	1652	78.3	21.7	74.9	25.1	77.9	22.1
	USD 75 k–USD 99,999	1016	84.5	15.5	80.5	19.5	83.2	16.8
	USD 100,000+	1204	85.7	14.3	81.9	18.1	84.8	15.2
	*p*-value		0.001		0.001		0.001	
Hispanic	Yes	1386	72.4	27.6	68.6	31.4	72.9	27.1
	No	7592	77.2	22.8	73.7	26.3	76.3	23.7
	*p*-value		0.001		0.001		0.006	
Race White	Yes	6677	76.8	23.2	72.9	27.1	75.9	24.1
	No	2302	75.5	24.5	72.8	27.2	75.4	24.6
	*p*-value		0.203		0.871		0.607	
Race Black	Yes	1224	75.4	24.6	72.5	27.5	74.7	25.3
	No	7754	76.6	23.4	72.9	27.1	75.9	24.1
	*p*-value		0.355		0.765		0.333	

GAD-2, Generalized Anxiety Disorder two-item assessment of anxiety; PHQ-2, Patient Health Questionnaire two-item assessment of depressive symptoms; PHQ-4, Patient Health Questionnaire four-item assessment of anxiety and depressive symptoms; Wt, weighted.

**Table 4:** Spearman correlation results between frequency of anxiety/depressive symptoms and COVID-19 impact or risk factors during the pandemic.

COVID-19 Impacts and Risk Factors	N	Spearman Rho
Sick for 3 days or longer when you thought you might have COVID-19	7984	0.217 **
Ever diagnosed with COVID-19	8987	0.080 **
Other household member (but not you) diagnosed with COVID-19	8987	0.079 **
Concerned about the spread of COVID-19 within your community	8969	0.132 **
Worried someone in your immediate family might catch COVID-19	8350	0.199 **
How likely do you think it is that you personally will get sick with COVID-19	8522	0.169 **
Likelihood of getting sick from COVID-19 on a scale of 0 to 100%	8569	0.188 **
Employer terminated or permanently laid off	5683	0.150 **
Employer furloughed or temporarily laid off	5683	0.053 **
Employer reduced your work hours	5683	0.112 **
Salary or hourly rate reduced	4427	0.078 **
None of the above employment changes	5683	−0.164 **
Compared to the end of 2019, how had your household income changes	6851	−0.156 **
Difficult to pay mortgage or rent	7927	0.318 **
Worried whether our food would run out before we got money to buy more	2961	−0.331 **
Food we bought just did not last, and we didn’t have money to get more	2957	−0.322 **
Overall how much if any has your life been disrupted by COVID-19	8967	0.163 **
How worried that your local hospitals did not have resources to treat all patients	8972	0.180 **
Have an underlying health condition that would increase risk of dying from COVID-19	8522	0.143 **

** *p* < 0.01.

**Table 5:** Linear, negative binomial, and zero-inflated regression results predicting PHQ-4 scores in Model 1: Demographics; n = 8840.

		Estimate (95% Confidence Interval)			
		Linear	Neg Bin	Zero ^1^	Adj Mean PHQ-4 Score
Intercept		1.33	1.03	−0.28	
		(1.08, 1.59)	(0.96, 1.11)	(−0.47, −0.09)	
Dispersion			0.25		
			(0.23, 0.28)		
Gender	Male	−0.56	−0.07	0.4	3.03
		(−0.70, −0.42)	(−0.11, −0.03)	(0.29, 0.51)	(2.93, 3.14)
	Female	Ref	Ref	Ref	3.6
					(3.51, 3.7)
Age group	18–24	2.31	0.33	−1.69	4.17
		(2.04, 2.58)	(0.26, 0.40)	(−1.94, −1.44)	(3.96, 4.38)
	25–34	2.19	0.34	−1.44	4.07
		(1.95, 2.43)	(0.27, 0.41)	(−1.64, −1.24)	(3.90, 4.24)
	35–49	1.85	0.36	−0.93	3.72
		(1.63, 2.07)	(0.29, 0.42)	(−1.08, −0.77)	(3.58, 3.86)
	50–64	1.13	0.25	−0.52	2.98
		(0.91, 1.35)	(0.18, 0.31)	(−0.68, −0.37)	(2.84, 3.11)
	65+	Ref	Ref	Ref	1.9
					(1.77, 2.04)
Race/ethnicity	Hispanic	−0.19	−0.05	−0.02	3.3
		(−0.37, −0.01)	(−0.10, −0.01)	(−0.17, 0.13)	(3.14, 3.45)
	NH White	Ref	Ref	Ref	3.45
					(3.35, 3.54)
	NH Black	−0.70	−0.11	0.35	2.8
		(−0.95, −0.46)	(−0.18, −0.04)	(0.16, 0.54)	(2.58, 3.01)
	NH other	−0.06	0.01	0.14	3.35
		(−0.33, 0.20)	(−0.06, 0.08)	(−0.08, 0.35)	(3.09, 3.62)
Educational attainment	Less than HS	1.02	0.25	−0.14	4.03
		(0.70, 1.34)	(0.17, 0.32)	(−0.41, 0.14)	(3.70, 4.37)
	HS Grad	0.31	0.14	0.16	3.36
		(0.12, 0.50)	(0.09, 0.19)	(0.02, 0.31)	(3.21, 3.50)
	Some College	0.45	0.13	−0.06	3.53
		(0.27, 0.62)	(0.08, 0.17)	(−0.20, 0.07)	(3.40, 3.65)
	College Grad	Ref	Ref	Ref	3.05
					(2.93, 3.17)
Household income	<USD 25,000	1.34	0.31	−0.34	4.05
		(1.09, 1.59)	(0.24, 0.37)	(−0.53, −0.15)	(3.87, 4.23)
	USD 25 k–USD 34,999	1.01	0.2	−0.45	3.74
		(0.75, 1.28)	(0.14, 0.27)	(−0.66, −0.24)	(3.55, 3.94)
	USD 35 k–USD 49,999	0.66	0.19	−0.07	3.38
		(0.4, 0.91)	(0.12, 0.26)	(−0.27, 0.12)	(3.19, 3.57)
	USD 50 k–USD 74,999	0.57	0.16	−0.09	3.29
		(0.33, 0.8)	(0.1, 0.22)	(−0.27, 0.09)	(3.13, 3.45)
	USD 75 k–USD 99,999	0.09	0.03	0	2.81
		(−0.16, 0.35)	(−0.04, 0.10)	(−0.19, 0.20)	(2.64, 2.99)
	USD 100,000+	Ref	Ref	Ref	2.74
					(2.58, 2.90)

PHQ-4, Patient Health Questionnaire four-item assessment of anxiety and depressive symptoms; Neg Bin, negative binomial; Adj, adjusted; Ref, reference group; NH, non-Hispanic; HS, high school. ^1^ Zero column displays results from zero-inflated negative binomial regression models.

**Table 6:** Linear, negative binomial, and zero-inflated regression results predicting PHQ-4 scores in Model 2: COVID-19 Illness Stressors; n = 8557.

		Estimate (95% Confidence Interval)			
		Linear	Neg Bin	Zero ^1^	Adj Mean PHQ-4 Score
HH COVID-19 Status	Respondent had COVID-19	1.96	0.24	−1.41	5.03
		(1.4, 2.52)	(0.09, 0.38)	(−1.93, −0.9)	(4.34, 5.72)
	Family member had COVID-19	1.48	0.21	−1.10	4.75
		(0.96, 2.00)	(0.08, 0.34)	(−1.63, −0.57)	(4.14, 5.37)
	Respondent and family member had COVID-19	2.29	0.27	−1.63	5.29
		(1.69, 2.90)	(0.12, 0.42)	(−2.23, −1.03)	(4.53, 6.06)
	No COVID-19 in HH	Ref	Ref	Ref	3.2
					(3.12, 3.28)
Worried that someone in family might catch COVID-19?	Very worried	0.7	0.12	−0.41	3.58
		(0.31, 1.08)	(0.01, 0.23)	(−0.71, −0.10)	(3.42, 3.73)
	Somewhat worried	0.28	0.04	−0.15	3.11
		(−0.08, 0.63)	(−0.06, 0.14)	(−0.43, 0.12)	(2.98, 3.24)
	Not too worried	0.07	0	−0.02	2.88
		(−0.26, 0.41)	(−0.1, 0.1)	(−0.28, 0.23)	(2.69, 3.06)
	Not at all worried	Ref	Ref	Ref	2.87
					(2.55, 3.18)
Concerned about spread of COVID-19 in community	Very concerned	−0.08	−0.05	−0.04	3.31
		(−0.35, 0.19)	(−0.13, 0.02)	(−0.26, 0.19)	(3.18, 3.44)
	Concerned	−0.14	−0.06	−0.02	3.28
		(−0.38, 0.09)	(−0.12, 0.01)	(−0.21, 0.17)	(3.15, 3.41)
	Not very concerned	Ref	Ref	Ref	3.45
					(3.23, 3.67)
Likelihood of personally getting sick with COVID-19	Very likely	0.23	0.02	0.07	3.27
		(−0.16, 0.61)	(−0.08, 0.12)	(−0.27, 0.40)	(2.94, 3.60)
	Somewhat likely	0.08	−0.06	−0.32	3.34
		(−0.19, 0.35)	(−0.14, 0.01)	(−0.54, −0.10)	(3.20, 3.49)
	Not too likely	−0.01	−0.09	−0.27	3.21
		(−0.24, 0.21)	(−0.16, −0.03)	(−0.44, −0.09)	(3.10, 3.32)
	Not at all likely	Ref	Ref	Ref	3.28
					(3.05, 3.51)
Likelihood of personally getting sick with COVID-19	1–10 Scale	0.07	0.01	−0.06	N/A
		(0.04, 0.10)	(0.00, 0.02)	(−0.09, −0.03)	
Health condition increasing risk of COVID-19 death	Yes	0.98	0.14	−0.64	3.91
		(0.83, 1.14)	(0.10, 0.18)	(−0.77, −0.50)	(3.78, 4.05)
	No	Ref	Ref	Ref	3.28
					(3.05, 3.32)
How much life disrupted by COVID-19	A lot	1.47	0.18	−1.12	3.77
		(1.07, 1.87)	(0.05, 0.30)	(−1.42, −0.82)	(3.64, 3.89)
	Moderate amount	0.89	0.06	−0.87	3.17
		(0.49, 1.28)	(−0.07, 0.19)	(−1.17, −0.57)	(3.06, 3.28)
	Only a little	0.65	0.07	−0.53	2.93
		(0.24, 1.06)	(−0.06, 0.20)	(−0.84, −0.22)	(2.76, 3.10)
	Not at all	Ref	Ref	Ref	2.24
					(1.88, 2.61)
Worried local hospitals will not have resources to treat COVID-19 patients	Very worried	0.36	0.07	−0.18	3.54
		(0.04, 0.68)	(−0.02, 0.15)	(−0.44, 0.07)	(3.39, 3.70)
	Somewhat worried	0.06	−0.01	−0.18	3.27
		(−0.23, 0.35)	(−0.10, 0.07)	(−0.41, 0.05)	(3.15, 3.40)
	Not too worried	0.01	−0.02	−0.09	3.2
		(−0.27, 0.29)	(−0.10, 0.07)	(−0.30, 0.12)	(3.04, 3.35)
	Not at all worried	Ref	Ref	Ref	3.17
					(2.90, 3.44)

PHQ-4, Patient Health Questionnaire four-item assessment of anxiety and depressive symptoms; Neg Bin, negative binomial; Adj, adjusted; HH, household; Ref, reference group; N/A, not applicable. ^1^ Zero column displays results from a zero-inflated negative binomial distribution regression model.

**Table 7:** Linear, negative binomial, and zero-inflated regression results predicting PHQ-4 scores in Model 3: Employment Stressors; n = 8515.

		Estimate (95% Confidence Interval)			
		Linear	Neg Bin	Zero ^1^	Adj Mean PHQ-4 Score
Employment changes	Not in labor force	0.72 (0.54, 0.90)	0.13 (0.09, 0.18)	−0.33 (−0.48, −0.18)	N/A
	Terminated	1.02 (0.74, 1.31)	0.15 (0.08, 0.22)	−0.69 (−0.98, −0.39)	3.95 (3.67, 4.22)
	Furloughed	0.53 (0.28, 0.78)	0.10 (0.03, 0.16)	−0.29 (−0.50, −0.07)	3.48 (3.25, 3.70)
	Pay/hours reduced	0.52 (0.30, 0.74)	0.06 (0.01, 0.12)	−0.49 (−0.69, −0.29)	3.50 (3.31, 3.68)
	No change in pay or hours	Ref	Ref	Ref	2.93 (2.81, 3.05)

PHQ-4, Patient Health Questionnaire four-item assessment of anxiety and depressive symptoms; Neg Bin, negative binomial; Adj, adjusted; N/A, not applicable; Ref, reference group. ^1^ Zero column displays results from a zero-inflated negative binomial distribution regression model.

**Table 8:** Linear, negative binomial, and zero-inflated regression results predicting PHQ-4 scores in Model 4: Additional COVID-19 Stressors; n = 7512.

		Estimate (95% Confidence Interval)			
		Linear	Neg Bin	Zero ^1^	Adj Mean PHQ-4 Score
Sick for 3+ days and might have COVID-19	Yes	0.82	0.09	−0.72	3.95
		(0.64, 0.99)	(0.05, 0.13)	(−0.91, −0.53)	(3.79, 4.12)
	No	Ref	Ref	Ref	3.11
					(3.03, 3.2)
Difficulty paying rent or mortgage	No rent/mortgage	Ref	Ref	Ref	3.44
					(3.19, 3.69)
	No difficulty	−0.73	−0.18	0.26	2.69
		(−0.98, −0.48)	(−0.24, −0.11)	(0.05, 0.46)	(2.58, 2.79)
	Little difficulty	−0.05	−0.06	−0.33	3.47
		(−0.33, 0.24)	(−0.14, 0.01)	(−0.58, −0.08)	(3.3, 3.64)
	Some difficulty	0.67	0.09	−0.42	4.12
		(0.38, 0.97)	(0.02, 0.16)	(−0.68, −0.16)	(3.91, 4.33)
	A lot of difficulty	1	0.09	−0.87	4.64
		(0.58, 1.41)	(−0.04, 0.23)	(−1.20, −0.55)	(4.38, 4.90)

PHQ-4, Patient Health Questionnaire four-item assessment of anxiety and depressive symptoms; Neg Bin, negative binomial; Adj, adjusted; Ref, reference group. ^1^ Zero column displays results from a zero-inflated negative binomial distribution regression model.

## Data Availability

The raw data supporting the conclusions of this article will be made available by the authors on request.

## Appendix A

**Table A1:** Negative binomial and zero-inflated regression results from Model 1: Demographics and Model 2: Stressors 1 predicting PHQ-4 scores.

		Model 1: Demographics		Model 2: Stressors 1	
		Mar–Nov (n = 8840)		Mar–Nov (n = 8557)	
		Estimate (95% Confidence Interval)			
		Neg Bin	Zero ^1^	Neg Bin	Zero ^1^
Intercept		1.03	−0.28	0.86	1.76
		(0.96, 1.11)	(−0.47, −0.09)	(0.7, 1.02)	(1.38, 2.15)
Dispersion		0.25		0.22	
		(0.23, 0.28)		(0.21, 0.25)	
Gender	Male	−0.07	0.4	−0.05	0.35
		(−0.11, −0.03)	(0.29, 0.51)	(−0.09, −0.02)	(0.23, 0.46)
	Female	Ref	Ref	Ref	Ref
Age group	18–24	0.33	−1.69	0.38	−1.88
		(0.26, 0.40)	(−1.94, −1.44)	(0.31, 0.46)	(−2.14, −1.62)
	25–34	0.34	−1.44	0.37	−1.70
		(0.27, 0.41)	(−1.64, −1.24)	(0.31, 0.44)	(−1.91, −1.48)
	35–49	0.36	−0.93	0.36	−1.08
		(0.29, 0.42)	(−1.08, −0.77)	(0.30, 0.43)	(−1.25, −0.90)
	50–64	0.25	−0.52	0.24	−0.57
		(0.18, 0.31)	(−0.68, −0.37)	(0.17, 0.31)	(−0.74, −0.41)
	65+	Ref	Ref	Ref	Ref
Race/ethnicity	Hispanic	−0.05	−0.02	−0.09	0.1
		(−0.10, −0.01)	(−0.17, 0.13)	(−0.14, −0.05)	(−0.06, 0.26)
	NH White	Ref	Ref	Ref	Ref
	NH Black	−0.11	0.35	−0.12	0.41
		(−0.18, −0.04)	(0.16, 0.54)	(−0.19, −0.05)	(0.21, 0.62)
	NH other	0.01	0.14	−0.03	0.23
		(−0.06, 0.08)	(−0.08, 0.35)	(−0.09, 0.04)	(0.01, 0.46)
Educational attainment	Less than HS	0.25	−0.14	0.23	−0.26
		(0.17, 0.32)	(−0.41, 0.14)	(0.15, 0.30)	(−0.56, 0.03)
	HS Grad	0.14	0.16	0.15	0.03
		(0.09, 0.19)	(0.02, 0.31)	(0.10, 0.20)	(−0.13, 0.18)
	Some College	0.13	−0.06	0.12	−0.13
		(0.08, 0.17)	(−0.20, 0.07)	(0.07, 0.17)	(−0.27, 0.02)
	College Grad	Ref	Ref	Ref	Ref
Household income	<USD 25,000	0.31	−0.34	0.28	−0.31
		(0.24, 0.37)	(−0.53, −0.15)	(0.21, 0.34)	(−0.52, −0.10)
	USD 25 k–USD 34,999	0.2	−0.45	0.19	−0.40
		(0.14, 0.27)	(−0.66, −0.24)	(0.12, 0.25)	(−0.62, −0.17)
	USD 35 k–USD 49,999	0.19	−0.07	0.17	−0.02
		(0.12, 0.26)	(−0.27, 0.12)	(0.11, 0.24)	(−0.23, 0.19)
	USD 50 k–USD 74,999	0.16	−0.09	0.14	−0.05
		(0.10, 0.22)	(−0.27, 0.09)	(0.08, 0.21)	(−0.24, 0.14)
	USD 75 k–USD 99,999	0.03	0	0.03	0
		(−0.04, 0.10)	(−0.19, 0.20)	(−0.04, 0.10)	(−0.20, 0.21)
	USD 100,000+	Ref	Ref	Ref	Ref
HH COVID-19 Status	Respondent had COVID-19			0.24	−1.41
				(0.09, 0.38)	(−1.93, −0.90)
	Family Member had COVID-19			0.21	−1.10
				(0.08, 0.34)	(−1.63, −0.57)
	Respondent and Family Member had COVID-19			0.27	−1.63
				(0.12, 0.42)	(−2.23, −1.03)
	No COVID-19 in HH			Ref	Ref
Worried that someone in family might catch COVID-19?	Very worried			0.12	−0.41
				(0.01, 0.23)	(−0.71, −0.10)
	Somewhat worried			0.04	−0.15
				(−0.06, 0.14)	(−0.43, 0.12)
	Not too worried			0	−0.02
				(−0.10, 0.10)	(−0.28, 0.23)
	Not at all worried			Ref	Ref
Likelihood of personally getting sick with COVID-19	Very likely			0.02	0.07
				(−0.08, 0.12)	(−0.27, 0.40)
	Somewhat likely			−0.06	−0.32
				(−0.14, 0.01)	(−0.54, −0.10)
	Not too likely			−0.09	−0.27
				(−0.16, −0.03)	(−0.44, −0.09)
	Not at all likely			Ref	Ref
Likelihood of personally getting sick with COVID-19	1–10 Scale			0.01	−0.06
				(0.00, 0.02)	(−0.09, −0.03)
Health condition increasing risk of COVID-19 death	Yes			0.14	−0.64
				(0.10, 0.18)	(−0.77, −0.50)
How much life disrupted by COVID-19	A lot			0.18	−1.12
				(0.05, 0.30)	(−1.42, −0.82)
	Moderate amount			0.06	−0.87
				(−0.07, 0.19)	(−1.17, −0.57)
	Only a little			0.07	−0.53
				(−0.06, 0.20)	(−0.84, −0.22)
	Not at all			Ref	Ref
Worried local hospitals will not have resources to treat COVID-19 patients	Very worried			0.07	−0.18
				(−0.02, 0.15)	(−0.44, 0.07)
	Somewhat worried			−0.01	−0.18
				(−0.10, 0.07)	(−0.41, 0.05)
	Not too worried			−0.02	−0.09
				(−0.10, 0.07)	(−0.30, 0.12)
	Not at all worried			Ref	Ref

PHQ-4, Patient Health Questionnaire four-item assessment of anxiety and depressive symptoms; Neg Bin, negative binomial; Ref, reference group; NH, non-Hispanic; HS, high school; HH, household. ^1^ Zero columns display results from zero-inflated negative binomial distribution regression models.

**Table A2:** Negative binomial and zero-inflated regression results from Model 3: Employment and Model 4: Stressors 2 predicting PHQ-4 scores.

		Model 3: Employment		Model 4: Stressors 2	
		Mar–Nov (n = 8515)		Apr–Nov (n = 7512)	
		Estimate (95% Confidence Interval)			
		Neg Bin	Zero ^1^	Neg Bin	Zero ^1^
Intercept		0.8	1.87	1.06	1.43
		(0.64, 0.96)	(1.48, 2.26)	(0.89, 1.24)	(0.98, 1.88)
Dispersion		0.22		0.2	
		(0.20, 0.24)		(0.18, 0.22)	
Gender	Male	−0.05	0.34	−0.05	0.37
		(−0.09, −0.01)	(0.22, 0.45)	(−0.09, −0.01)	(0.24, 0.49)
	Female	Ref	Ref	Ref	Ref
Age group	18–24	0.4	−1.90	0.35	−1.82
		(0.33, 0.48)	(−2.17, −1.64)	(0.27, 0.43)	(−2.11, −1.54)
	25–34	0.4	−1.75	0.35	−1.54
		(0.33, 0.47)	(−1.97, −1.52)	(0.28, 0.42)	(−1.78, −1.3)
	35–49	0.39	−1.14	0.34	−0.98
		(0.33, 0.46)	(−1.32, −0.95)	(0.27, 0.42)	(−1.18, −0.78)
	50–64	0.26	−0.64	0.23	−0.57
		(0.19, 0.33)	(−0.81, −0.46)	(0.16, 0.3)	(−0.76, −0.38)
	65+	Ref	Ref	Ref	Ref
Race/ethnicity	Hispanic	−0.09	0.1	−0.10	0.12
		(−0.14, −0.05)	(−0.07, 0.26)	(−0.15, −0.05)	(−0.05, 0.30)
	NH White	Ref	Ref	Ref	Ref
	NH Black	−0.11	0.42	−0.11	0.39
		(−0.18, −0.04)	(0.21, 0.62)	(−0.18, −0.04)	(0.17, 0.61)
	NH other	−0.02	0.24	−0.04	0.28
		(−0.09, 0.04)	(0.02, 0.47)	(−0.11, 0.03)	(0.03, 0.52)
Educational attainment	Less than HS	0.21	−0.19	0.17	−0.04
		(0.13, 0.28)	(−0.49, 0.11)	(0.09, 0.25)	(−0.37, 0.29)
	HS Grad	0.13	0.07	0.12	0.1
		(0.08, 0.18)	(−0.09, 0.23)	(0.06, 0.17)	(−0.07, 0.27)
	Some College	0.1	−0.08	0.08	−0.09
		(0.06, 0.15)	(−0.23, 0.06)	(0.03, 0.13)	(−0.25, 0.07)
	College Grad	Ref	Ref	Ref	Ref
Household income	<USD 25,000	0.24	−0.21	0.15	−0.12
		(0.17, 0.3)	(−0.43, 0.00)	(0.08, 0.22)	(−0.35, 0.12)
	USD 25 k–USD 34,999	0.16	−0.33	0.09	−0.17
		(0.09, 0.23)	(−0.55, −0.10)	(0.02, 0.16)	(−0.41, 0.07)
	USD 35 k–USD 49,999	0.15	0.04	0.09	0.19
		(0.09, 0.22)	(−0.17, 0.24)	(0.02, 0.16)	(−0.04, 0.41)
	USD 50 k–USD 74,999	0.12	−0.02	0.08	0.1
		(0.06, 0.19)	(−0.21, 0.17)	(0.02, 0.15)	(−0.1, 0.30)
	USD 75 k–USD 99,999	0.02	0.02	0	0.11
		(−0.05, 0.09)	(−0.19, 0.22)	(−0.07, 0.07)	(−0.11, 0.33)
	USD 100,000+	Ref	Ref	Ref	Ref
HH COVID-19 Status	Respondent had COVID-19	0.23	−1.29	0.13	−0.77
		(0.09, 0.37)	(−1.81, −0.76)	(−0.02, 0.27)	(−1.33, −0.21)
	Family Member had COVID-19	0.22	−1.05	0.17	−0.72
		(0.08, 0.35)	(−1.58, −0.52)	(0.04, 0.3)	(−1.25, −0.18)
	Respondent and Family Member had COVID-19	0.28	−1.60	0.18	−0.94
		(0.13, 0.42)	(−2.2, −1)	(0.02, 0.33)	(−1.55, −0.33)
	No COVID-19 in HH	Ref	Ref	Ref	Ref
Worried that someone in family might catch COVID-19?	Very worried	0.13	−0.39	0.12	−0.26
		(0.02, 0.23)	(−0.69, −0.08)	(0.01, 0.23)	(−0.59, 0.07)
	Somewhat worried	0.05	−0.13	0.05	−0.08
		(−0.06, 0.15)	(−0.41, 0.15)	(−0.06, 0.15)	(−0.38, 0.22)
	Not too worried	0	−0.01	−0.01	0.08
		(−0.10, 0.10)	(−0.27, 0.25)	(−0.11, 0.09)	(−0.2, 0.35)
	Not at all worried	Ref	Ref	Ref	Ref
Likelihood of personally getting sick with COVID-19	Very likely	0.02	0.09	−0.04	0.25
		(−0.08, 0.11)	(−0.25, 0.42)	(−0.15, 0.06)	(−0.12, 0.61)
	Somewhat likely	−0.06	−0.31	−0.08	−0.20
		(−0.13, 0.01)	(−0.54, −0.09)	(−0.16, −0.01)	(−0.44, 0.05)
	Not too likely	−0.09	−0.26	−0.09	−0.26
		(−0.15, −0.02)	(−0.44, −0.08)	(−0.15, −0.02)	(−0.45, −0.07)
	Not at all likely	Ref	Ref	Ref	Ref
Likelihood of personally getting sick with COVID-19	1–10 Scale	0.01	−0.06	0.01	−0.06
		(0.00, 0.02)	(−0.10, −0.03)	(0.00, 0.02)	(−0.10, −0.03)
Health condition increasing risk of COVID-19 death	Yes	0.13	−0.61	0.1	−0.57
		(0.09, 0.17)	(−0.75, −0.47)	(0.06, 0.15)	(−0.71, −0.42)
How much life disrupted by COVID-19	A lot	0.16	−1.00	0.09	−0.87
		(0.03, 0.29)	(−1.31, −0.69)	(−0.04, 0.23)	(−1.20, −0.55)
	Moderate amount	0.05	−0.78	0.01	−0.66
		(−0.08, 0.18)	(−1.09, −0.48)	(−0.12, 0.14)	(−0.98, −0.34)
	Only a little	0.06	−0.47	0.03	−0.39
		(−0.07, 0.20)	(−0.78, −0.16)	(−0.10, 0.17)	(−0.72, −0.07)
	Not at all	Ref	Ref	Ref	Ref
Worried local hospitals will not have resources to treat COVID-19 patients	Very worried	0.06	−0.19	0.03	−0.03
		(−0.03, 0.15)	(−0.45, 0.07)	(−0.06, 0.12)	(−0.3, 0.25)
	Somewhat worried	−0.02	−0.21	−0.05	−0.21
		(−0.10, 0.07)	(−0.44, 0.02)	(−0.13, 0.04)	(−0.46, 0.03)
	Not too worried	−0.02	−0.10	−0.05	−0.12
		(−0.10, 0.06)	(−0.32, 0.11)	(−0.13, 0.03)	(−0.35, 0.10)
	Not at all worried	Ref	Ref	Ref	Ref
Employment changes	Not in labor force	0.13	−0.33	0.11	−0.25
		(0.09, 0.18)	(−0.48, −0.18)	(0.06, 0.16)	(−0.42, −0.09)
	Terminated	0.15	−0.69	0.05	−0.32
		(0.08, 0.22)	(−0.98, −0.39)	(−0.02, 0.12)	(−0.63, −0.02)
	Furloughed	0.1	−0.29	0.03	−0.14
		(0.03, 0.16)	(−0.5, −0.07)	(−0.04, 0.10)	(−0.38, 0.09)
	Pay reduced	0.06	−0.49	0	−0.30
		(0.01, 0.12)	(−0.69, −0.29)	(−0.06, 0.06)	(−0.52, −0.08)
	No change in pay or hours	Ref	Ref	Ref	Ref
Sick for 3+ days and might have COVID	Yes			0.09	−0.72
				(0.05, 0.13)	(−0.91, −0.53)
Difficulty paying rent or mortgage	No rent/mortgage			Ref	Ref
	No difficulty			−0.18	0.26
				(−0.24, −0.11)	(0.05, 0.46)
	Little difficulty			−0.06	−0.33
				(−0.14, 0.01)	(−0.58, −0.08)
	Some difficulty			0.09	−0.42
				(0.02, 0.16)	(−0.68, −0.16)
	A lot of difficulty			0.09	−0.87
				(−0.04, 0.23)	(−1.20, −0.55)

PHQ-4, Patient Health Questionnaire four-item assessment of anxiety and depressive symptoms; Neg Bin, negative binomial; NH, non-Hispanic; HS, high school; HH, household; Ref, reference group. ^1^ Zero column displays results from a zero-inflated negative binomial distribution regression model.
